# Crystal structures of 6-chloro­indan-1-one and 6-bromo­indan-1-one exhibit different inter­molecular packing inter­actions

**DOI:** 10.1107/S2056989016015371

**Published:** 2016-10-07

**Authors:** Alessio Caruso, Benjamin Blair, Joseph M. Tanski

**Affiliations:** a Department of Chemistry, Vassar College, Poughkeepsie, NY 12604, USA

**Keywords:** crystal structure, haloindanones, π-stacking, C—H⋯*X* inter­actions

## Abstract

The two title compounds are analogs of 1-indanone that are substituted at the 6-position with chlorine and bromine. Although very similar in mol­ecular structure, the crystal structures are not isomorphous and reveal that 6-chloro­indan-1-one, C_9_H_7_ClO (I), and 6-bromo­indan-1-one, C_9_H_7_BrO (II), exhibit unique inter­molecular packing motifs. The mol­ecules of the chloro analog (I) pack with a herringbone packing motif of C—H⋯O inter­actions, whereas the bromo derivative (II) packs with offset face-to-face π-stacking, C—H⋯O, C—H⋯Br and Br⋯O inter­actions. Compound (II) was refined as a two-component non-merohedral twin, BASF 0.0762 (5).

## Abstract 2

The structures of two haloindanones are reported and differences in their inter­molecular packing inter­actions are explored.

## Chemical context   

Halogenated derivatives of the common bicyclic organic framework 1-indanone have been shown to be useful in a variety of synthetic and biologically related applications (Ruiz *et al.*, 2004 ▸). A search of the Cambridge Structural Database (Version 5.31, September 2016 with updates; Groom *et al.*, 2016 ▸) returns four simple aryl­halide substituted 1-indanones, although several more are commercially available. The title compounds represent two analogs of 6-haloindan-1-one that are notably not isomorphous. In addition, they are not isomorphous with the fluorine derivative 6-fluoro­indan-1-one, which is one of the four that has previously been reported (Slaw & Tanski, 2014 ▸). In the chloro analog, 6-chloro­indan-1-one (I), the mol­ecules pack together *via* a series of C—H⋯O inter­actions. C—H⋯*X* inter­actions are common and have been discussed in the literature (Desiraju & Steiner, 1999 ▸), as well as specifically in the case of 1-indanone itself (Ruiz *et al.*, 2004 ▸). The bromo derivative 6-bromo­indan-1-one (II) packs with offset face-to-face π-stacking (Hunter & Saunders, 1990 ▸; Lueckheide *et al.*, 2013 ▸) and several different inter­molecular contacts including C—H⋯O, C—H⋯Br weak hydrogen bonds and Br⋯O inter­actions.
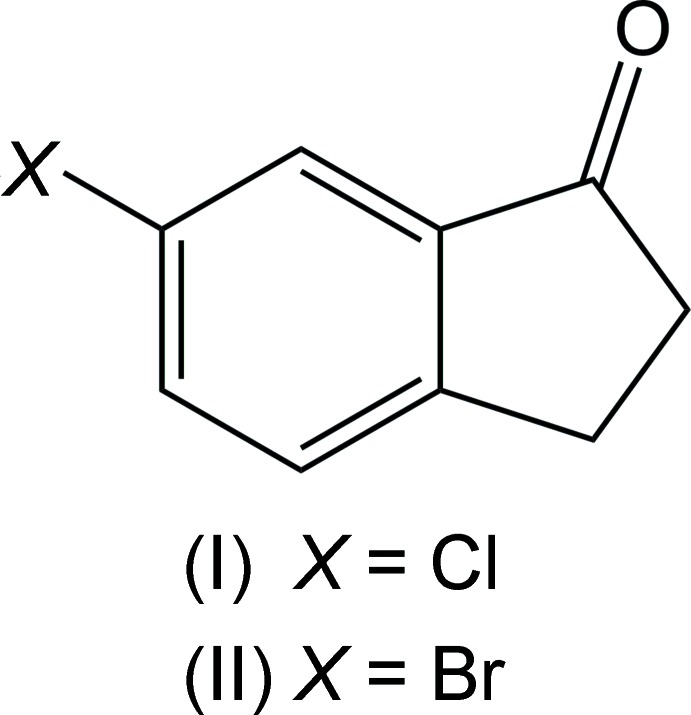


The compounds 6-chloro­indan-1-one (I) and 6-bromo­indan-1-one (II) may be synthesized by the microwave or ultrasound-aided ring closure of 4-chloro- or 4-bromo­benzene­propanoic acid, respectively, catalyzed by triflic acid in di­chloro­methane (Oliverio *et al.*, 2014 ▸). 6-Haloindan-1-ones have featured in the synthesis of biologically or pharmacologically active compounds. In recent examples, 6-chloro­indan-1-one (I) has been employed in the total synthesis of the anti­cancer natural product chartarin (Unzner *et al.*, 2016 ▸), and in the synthesis of triazole-quinoline derivatives that are acetyl­cholinesterase inhibitors relevant to the treatment of Alzheimer’s disease (Mantoani *et al.*, 2016 ▸). 6-Bromo­indan-1-one has been used as the starting material for the synthesis of small mol­ecules that inhibit cell entry by HIV-1 (Melillo *et al.*, 2016 ▸), and both 6-chloro­indan-1-one and 6-bromo­indan-1-one have been used as the starting material for the preparation of C-7 substituted 3,4-di­hydro­isoquinolin-1(*2H*)-one analogues that selectively inhibit unique poly-ADP-ribose polymerases (Morgan *et al.*, 2015 ▸).

## Structural commentary   

**Figure 1:**
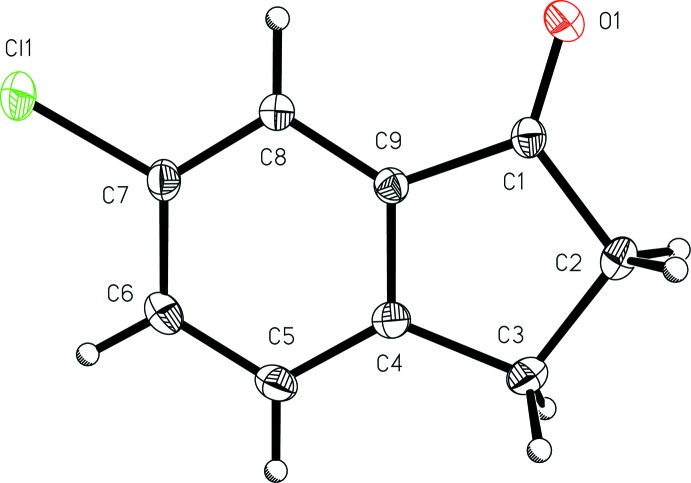
A view of 6-chloro­indan-1-one (I) with the atom-numbering scheme. Displacement ellipsoids are shown at the 50% probability level.

**Figure 2:**
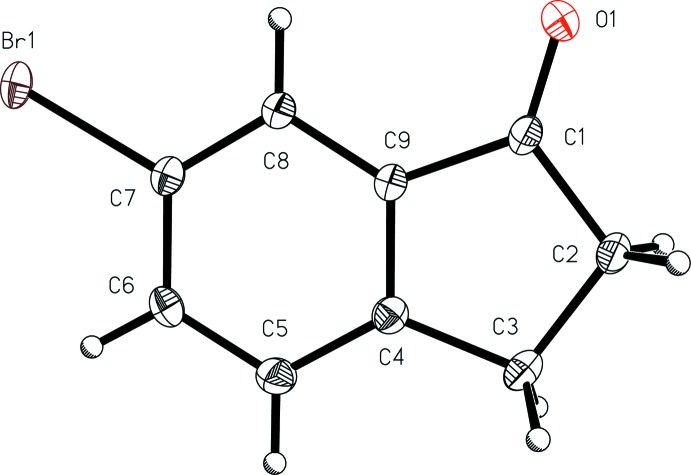
A view of 6-bromo­indan-1-one (II) with the atom-numbering scheme. Displacement ellipsoids are shown at the 50% probability level.

The mol­ecular features of 6-chloro­indan-1-one (I) (Fig. 1 ▸) and 6-bromo­indan-1-one (II) (Fig. 2 ▸) are similar to those reported for the analogous structure 6-fluoro­indan-1-one (Slaw & Tanski, 2014 ▸), although the analogues are not isomorphous and exhibit different inter­molecular packing. In the chloro derivative (I), the aryl C—Cl bond length, 1.7435 (11) Å, is similar to that found in the isomeric compound 5-chloro­indan-1-one [C—Cl = 1.735 (2) Å; Ruiz *et al.*, 2006 ▸]. The aryl C—Br bond length in the bromo analog (II), 1.907 (3) Å, is similar to that found in the isomeric compound 4-bromo­indan-1-one [1.894 (1) Å; Aldeborgh *et al.*, 2014 ▸]. The C=O bond lengths in 6-chloro­indan-1-one (I), 1.2200 (12) Å, and 6-bromo­indan-1-one (II), 1.216 (3) Å, are also very similar to those found in the other four reported structures of simple aryl­halide-substituted 1-indanones: 6-fluoro­indan-1-one, 1.2172 (13) Å (Slaw & Tanski, 2014 ▸); 5-fluoro­indan-1-one, 1.218 (2) Å (Garcia *et al.*,1995 ▸); 5-chloro­indan-1-one, 1.210 (3) Å (Ruiz *et al.*, 2006 ▸); 4-bromo­indan-1-one, 1.215 (2) Å (Aldeborgh *et al.*, 2014 ▸). These carbonyl C=O bond lengths are also similar to that found in the structure of the parent compound, 1-indanone, 1.217 (2) Å (Ruiz *et al.*, 2004 ▸). With the exception of the methyl­ene hydrogen atoms, both (I) and (II) are nearly planar, with r.m.s. deviations from the mean planes of all non-H atoms of 0.0460 and 0.0107 Å, respectively.

## Supra­molecular features   

**Figure 3:**
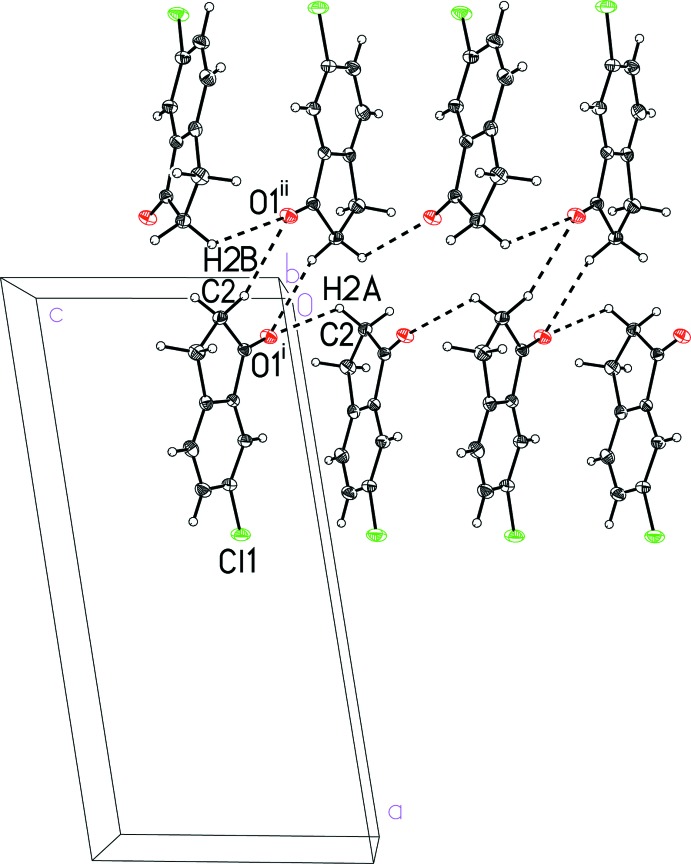
A view of the inter­molecular C—H⋯O contacts in 6-chloro­indan-1-one (I). See Table 1 ▸ for symmetry codes (i) and (ii). In this and subsequent figures the C—H⋯*X* inter­actions are shown as dashed lines.

**Figure 4:**
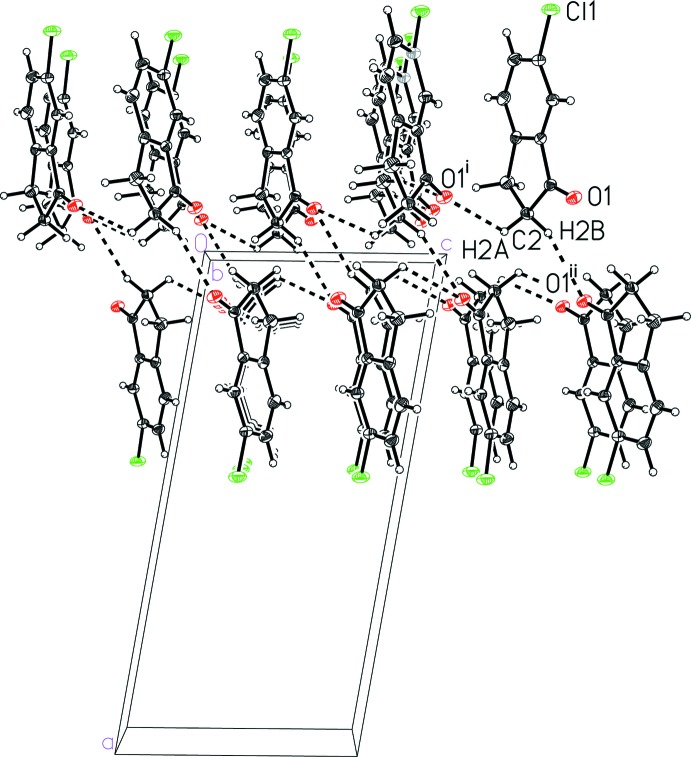
A view of the sheet structure in 6-chloro­indan-1-one (I) formed by C—H⋯O contacts. See Table 1 ▸ for symmetry codes (i) and (ii).

In the crystal structure of 6-chloro­indan-1-one (I), the mol­ecules pack together *via* van der Waals contacts, specifically C—H⋯O inter­actions, without any π-stacking. The C—H⋯O inter­actions (Fig. 3 ▸ and Table 1 ▸) connect the indanone oxygen atom with methyl­ene hydrogen atoms on neighboring mol­ecules into a two-mol­ecule-thick sheet parallel to the (100) plane (Fig. 4 ▸). These sheets further pack together without any notable inter­molecular contacts. The closest Cl⋯Cl contact between the sheets, 3.728 Å, is somewhat longer than the sum of the van der Waals radii of chlorine, 3.50 Å (Bondi, 1964 ▸).

**Figure 5:**
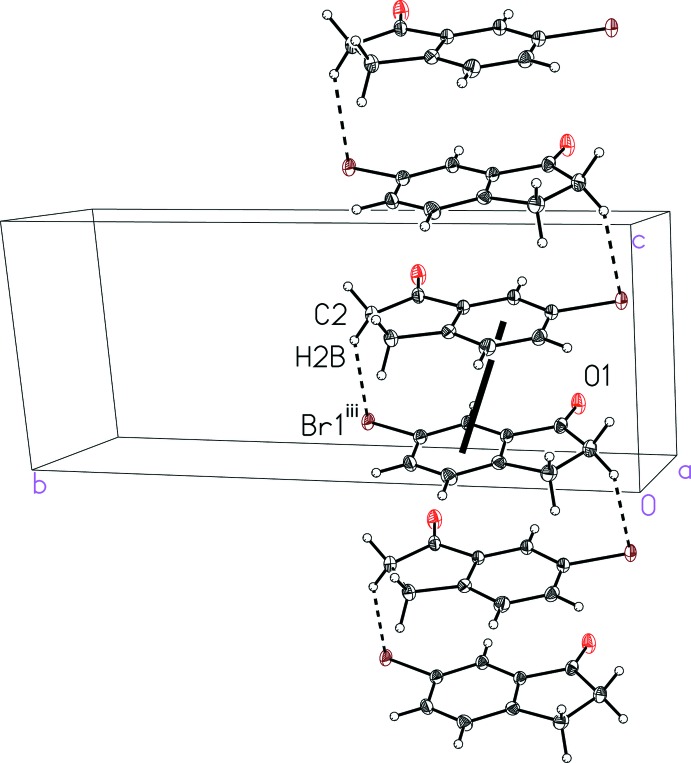
A view of the alternating offset face-to-face π-stacking and C—H⋯Br inter­action in 6-bromo­indan-1-one (II) with the thick black line indicating a centroid-to-centroid inter­action. See Table 2 ▸ for symmetry code (iii).

**Figure 6:**
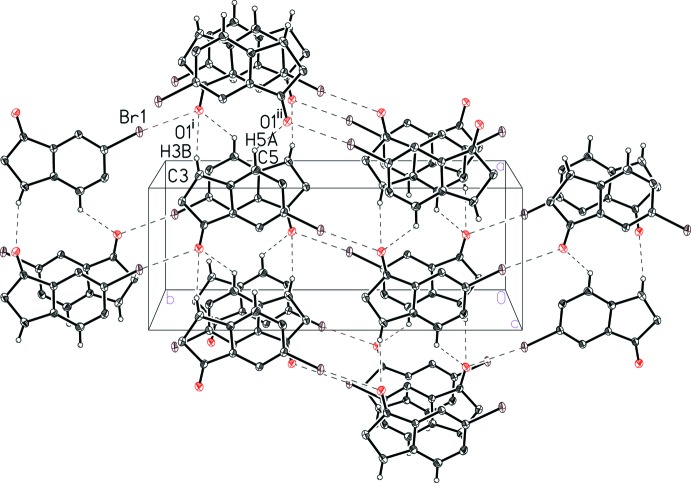
A view of the inter­molecular C—H⋯O and Br⋯O contacts (dashed lines) in 6-bromo­indan-1-one (II). See Table 2 ▸ for symmetry codes (i) and (ii).

The mol­ecular packing in the bromo analog, 6-bromo­indan-1-one (II), is distinct from that found in (I). The notable inter­molecular inter­actions observed include π-stacking, Br⋯O, C—H⋯O, and C—H⋯Br inter­actions. The offset face-to-face π-stacking can be seen to extend along the crystallographic *c* axis (Fig. 5 ▸), with the mol­ecules stacking in an alternating head-to-tail fashion featuring a C—H⋯Br inter­action with an H⋯Br distance of 3.05 Å (Fig. 5 ▸ and Table 2 ▸). The π-stacking is characterized by a centroid-to-centroid distance of 3.850 (3) Å, centroid-to-plane distances of 3.530 (2) and 3.603 (2) Å, and ring offsets of 1.358 (3) and 1.536 (3) Å that result in a plane-to-plane angle of 3.1 (1)°. The π-stacked chains of (II) are linked into a three-dimensional lattice by C—H⋯O inter­actions and a Br⋯O contact (Fig. 6 ▸ and Table 2 ▸). The Br⋯O contact, at a distance of 3.018 (2) Å, is slightly shorter than the sum of the van der Waals radii, 3.37 Å (Bondi 1964 ▸). This inter­action is even shorter than the Br⋯O contact in the isomeric 4-bromo­indan-1-one [3.129 (1) Å; Aldeborgh *et al.*, 2014 ▸].

## Database survey   

A survey of the Cambridge Structural Database reveals that in addition to the two structures reported here, there are four other simple aryl­halide-substituted 1-indanone structures known. These include 6-fluoro­indan-1-one (Slaw & Tanski, 2014 ▸), 5-fluoro­indan-1-one (Garcia *et al.*, 1995 ▸), 5-chloro­indan-1-one (Ruiz *et al.*, 2006 ▸) and 4-bromo­indan-1-one (Aldeborgh *et al.*, 2014 ▸). The crystal structure of 1-indanone itself was first reported in 1974 (Morin *et al.*, 1974 ▸) and was later described in a more detailed structural and spectroscopic analysis (Ruiz *et al.*, 2004 ▸).

## Synthesis and crystallization   

6-Chloro­indan-1-one (96%) and 6-bromo­indan-1-one (98%) were purchased from Aldrich Chemical Company, USA, and were used as received.

## Analytical data   

**6-Chloro­indan-1-one (I):** ^1^H NMR (Bruker Avance 300 MHz, CDCl_3_): δ 2.72 (*t*, 2 H, *J* = 5.9 Hz, C*H_2_*), 3.12 (*t*, 2H, *J* = 5.9 Hz, C*H_2_*), 7.42 (*d*, 1 H, *J_ortho_* = 8.2 Hz, C_ar­yl_ *H*), 7.53 (*dd*, 1H, *J_meta_* = 1.6 Hz, *J_ortho_* = 8.1 Hz, C_ar­yl_ *H*), 7.69 (*s*, 1 H, C_ar­yl_ *H*). ^13^C NMR (^13^C{^1^H}, 75.5 MHz, CDCl_3_): δ 25.37 (*C*H_2_), 36.57 (*C*H_2_), 123.45 (*C*_ar­yl_H), 127.85 (*C*_ar­yl_H), 133.63 (*C*_ar­yl_), 134.50 (*C*_ar­yl_H), 138.49 (*C*_ar­yl_), 153.07 (*C*_ar­yl_), 205.43 (*C*=O). IR (Thermo Nicolet iS50, KBr pellet, cm^−1^): 3391 (*w*), 3076 (*w*), 3051 (*w*), 2964 (*w*), 2935 (*w*), 1702 (*vs*, C=O *str*), 1595 (*w*), 1576 (*w*), 1466 (*m*), 1435 (*m*), 1409 (*m*), 1318 (*w*), 1285 (*w*), 1276 (*w*), 1250 (*m*), 1214 (*w*), 1187 (*m*), 1173 (*m*), 1115 (*m*), 1037 (*w*), 895 (*m*), 854 (*m*), 836 (*s*), 815 (*m*), 678 (*m*), 623 (*m*), 561 (*m*), 518 (*w*), 484 (*m*). GC/MS (Hewlett-Packard MS 5975/GC 7890): *M* ^+^ = 166 (calculated exact mass 166.02).

**6-Bromo­indan-1-one (II):** ^1^H NMR (Bruker Avance 300 MHz, CDCl_3_): δ 2.71 (*t*, 2 H, *J* = 5.8 Hz, C*H_2_*), 3.09 (*t*, 2H, *J* = 5.9 Hz, C*H_2_*), 7.37 (*d*, 1 H, *J_ortho_* = 8.1 Hz, C_ar­yl_ *H*), 7.65 (*dd*, 1H, *J_meta_* = 1.9 Hz, *J_ortho_* = 8.1 Hz, C_ar­yl_ *H*), 7.83 (*s*, 1 H, C_ar­yl_ *H*). ^13^C NMR (^13^C{^1^H}, 75.5 MHz, CDCl_3_): δ 25.37 (*C*H_2_), 36.34 (*C*H_2_), 121.35 (*C*_ar­yl_), 126.46 (*C*_ar­yl_H), 128.16 (*C*_ar­yl_H), 137.14 (*C*_ar­yl_H), 138.73 (*C*_ar­yl_), 153.47 (*C*_ar­yl_), 205.19 (*C*=O). IR (Thermo Nicolet iS50, ATR, cm^−1^): 3394 (*w*), 3066 (*w*), 2962 (*w*), 2925 (*w*), 1698 (*vs*, C=O *str*), 1598 (*w*), 1577 (*w*), 1468 (*w*), 1438 (*s*), 1417 (*w*), 1398 (*m*), 1322 (*w*), 1295 (*w*), 1279 (*w*), 1253 (*m*), 1238 (*m*), 1213 (*w*), 1191 (*s*), 1171 (*w*), 1112 (*m*), 1038 (*w*), 978 (*w*), 887 (*w*), 829 (*s*), 668 (*m*), 609 (*w*), 557 (*m*), 509 (*w*), 478 (*m*). GC/MS (Hewlett-Packard MS 5975/GC 7890): *M* ^+^ = 210 (calculated exact mass 209.97).

## Refinement   

Crystal data, data collection and structure refinement details are summarized in Table 3 ▸. After indexing with *Cell_Now* (Sheldrick, 2008 ▸), 6-bromo­indan-1-one (II) was refined as a two-component non-merohedral twin, BASF 0.0762 (5). Carbon-bound hydrogen atoms were included in calculated positions and refined using a riding model at C—H = 0.95 and 0.99 Å and *U*_iso_(H) = 1.2*U*_eq_(C) of the aryl and methyl­ene C atoms, respectively.

## Supplementary Material

Crystal structure: contains datablock(s) global, I, II. DOI: 10.1107/S2056989016015371/sj5508sup1.cif

Structure factors: contains datablock(s) I. DOI: 10.1107/S2056989016015371/sj5508Isup2.hkl

Structure factors: contains datablock(s) II. DOI: 10.1107/S2056989016015371/sj5508IIsup3.hkl

Supporting information file. DOI: 10.1107/S2056989016015371/sj5508Isup4.cml

Supporting information file. DOI: 10.1107/S2056989016015371/sj5508IIsup5.cml

CCDC references: 1507437, 1507436

Additional supporting information:  crystallographic information; 3D view; checkCIF report

## Figures and Tables

**Table 1:** Hydrogen-bond geometry (Å, °) for (I)

*D*—H⋯*A*	*D*—H	H⋯*A*	*D*⋯*A*	*D*—H⋯*A*
C2—H2*A*⋯O1^i^	0.99	2.56	3.1933 (15)	121
C2—H2*B*⋯O1^ii^	0.99	2.59	3.5448 (14)	161

Symmetry codes: (i) 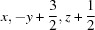; (ii) 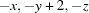.

**Table 2:** Hydrogen-bond geometry (Å, °) for (II)

*D*—H⋯*A*	*D*—H	H⋯*A*	*D*⋯*A*	*D*—H⋯*A*
C3—H3*B*⋯O1^i^	0.99	2.45	3.408 (4)	162
C5—H5*A*⋯O1^ii^	0.95	2.55	3.253 (4)	131
C2—H2*B*⋯Br1^iii^	0.99	3.05	3.898 (3)	145

Symmetry codes: (i) 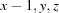; (ii) 
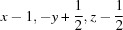
; (iii) 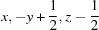.

**Table 3:** Experimental details

	(I)	(II)
Crystal data		
Chemical formula	C_9_H_7_ClO	C_9_H_7_BrO
*M*_r_	166.60	211.06
Crystal system, space group	Monoclinic, *P*2_1_/*c*	Monoclinic, *P*2_1_/*c*
Temperature (K)	125	125
*a*, *b*, *c* (Å)	16.319 (6), 6.024 (2), 7.745 (3)	6.489 (2), 17.101 (6), 7.224 (3)
β (°)	99.524 (5)	102.964 (5)
*V* (Å^3^)	750.9 (5)	781.2 (5)
*Z*	4	4
Radiation type	Mo *K*α	Mo *K*α
μ (mm^−1^)	0.44	5.19
Crystal size (mm)	0.28 × 0.25 × 0.14	0.40 × 0.21 × 0.05

Data collection		
Diffractometer	Bruker APEXII CCD	Bruker APEXII CCD
Absorption correction	Multi-scan (*SADABS*; Bruker, 2013 ▸)	Multi-scan (*TWINABS*; Bruker 2013 ▸)
*T*_min_, *T*_max_	0.84, 0.94	0.55, 0.78
No. of measured, independent and observed [*I* > 2σ(*I*)] reflections	18572, 2291, 2158	4453, 4453, 3600
*R*_int_	0.027	0.046
(sin θ/λ)_max_ (Å^−1^)	0.716	0.716

Refinement		
*R*[*F* ^2^ > 2σ(*F* ^2^)], *wR*(*F* ^2^), *S*	0.030, 0.083, 1.08	0.030, 0.152, 1.03
No. of reflections	2291	4453
No. of parameters	100	101
H-atom treatment	H-atom parameters constrained	H-atom parameters constrained
Δρ_max_, Δρ_min_ (e Å^−3^)	0.47, −0.23	1.15, −1.15

Computer programs: *APEX2* and *SAINT* (Bruker, 2013 ▸), *SHELXS* and *SHELXTL2014* (Sheldrick, 2008 ▸), *SHELXL2014/6* (Sheldrick, 2015 ▸), *OLEX2* (Dolomanov *et al.*, 2009 ▸) and *Mercury* (Macrae *et al.*, 2008 ▸).

## supplementary crystallographic information

### (I) 6-Chloroindan-1-one Crystal data

**Table:**

C_9_H_7_ClO	*F*(000) = 344
*M**_r_* = 166.60	*D*_x_ = 1.474 Mg m^−^^3^
Monoclinic, *P*2_1_/*c*	Mo *K*α radiation, λ = 0.71073 Å
*a* = 16.319 (6) Å	Cell parameters from 9875 reflections
*b* = 6.024 (2) Å	θ = 2.5–30.5°
*c* = 7.745 (3) Å	µ = 0.44 mm^−^^1^
β = 99.524 (5)°	*T* = 125 K
*V* = 750.9 (5) Å^3^	Block, colourless
*Z* = 4	0.28 × 0.25 × 0.14 mm

### (I) 6-Chloroindan-1-one Data collection

**Table:**

Bruker APEXII CCD diffractometer	2291 independent reflections
Radiation source: fine-focus sealed tube	2158 reflections with *I* > 2σ(*I*)
Graphite monochromator	*R*_int_ = 0.027
Detector resolution: 8.3333 pixels mm^-1^	θ_max_ = 30.6°, θ_min_ = 2.5°
φ and ω scans	*h* = −23→23
Absorption correction: multi-scan (SADABS; Bruker, 2013)	*k* = −8→8
*T*_min_ = 0.84, *T*_max_ = 0.94	*l* = −11→11
18572 measured reflections	

### (I) 6-Chloroindan-1-one Refinement

**Table:**

Refinement on *F*^2^	0 restraints
Least-squares matrix: full	Hydrogen site location: inferred from neighbouring sites
*R*[*F*^2^ > 2σ(*F*^2^)] = 0.030	H-atom parameters constrained
*wR*(*F*^2^) = 0.083	*w* = 1/[σ^2^(*F*_o_^2^) + (0.0439*P*)^2^ + 0.2902*P*] where *P* = (*F*_o_^2^ + 2*F*_c_^2^)/3
*S* = 1.08	(Δ/σ)_max_ = 0.001
2291 reflections	Δρ_max_ = 0.47 e Å^−^^3^
100 parameters	Δρ_min_ = −0.23 e Å^−^^3^

### (I) 6-Chloroindan-1-one Special details

**Table:**

Geometry. All esds (except the esd in the dihedral angle between two l.s. planes) are estimated using the full covariance matrix. The cell esds are taken into account individually in the estimation of esds in distances, angles and torsion angles; correlations between esds in cell parameters are only used when they are defined by crystal symmetry. An approximate (isotropic) treatment of cell esds is used for estimating esds involving l.s. planes.

### (I) 6-Chloroindan-1-one Fractional atomic coordinates and isotropic or equivalent isotropic displacement parameters (Å^2^)

**Table:**

	*x*	*y*	*z*	*U*_iso_*/*U*_eq_	
Cl1	0.44180 (2)	0.68505 (5)	0.30384 (4)	0.02625 (9)	
O1	0.09308 (5)	0.71579 (13)	0.08199 (10)	0.02037 (16)	
C1	0.12100 (5)	0.87485 (15)	0.17008 (11)	0.01444 (17)	
C2	0.07137 (6)	1.06661 (17)	0.22843 (12)	0.01704 (18)	
H2A	0.035	1.0136	0.3102	0.02*	
H2B	0.0363	1.1363	0.1262	0.02*	
C3	0.13555 (6)	1.23410 (17)	0.32015 (14)	0.02079 (19)	
H3A	0.1328	1.3753	0.254	0.025*	
H3B	0.1257	1.2653	0.4405	0.025*	
C4	0.21867 (6)	1.12199 (16)	0.32313 (12)	0.01535 (17)	
C5	0.29771 (6)	1.19687 (16)	0.39740 (13)	0.01873 (19)	
H5A	0.3046	1.3374	0.4536	0.022*	
C6	0.36615 (6)	1.06251 (17)	0.38780 (13)	0.01935 (19)	
H6A	0.4203	1.1114	0.4374	0.023*	
C7	0.35530 (6)	0.85462 (17)	0.30482 (13)	0.01715 (18)	
C8	0.27767 (6)	0.77850 (16)	0.22694 (12)	0.01593 (17)	
H8A	0.2708	0.6394	0.1685	0.019*	
C9	0.21000 (5)	0.91673 (15)	0.23876 (11)	0.01374 (17)	

### (I) 6-Chloroindan-1-one Atomic displacement parameters (Å^2^)

**Table:**

	*U*^11^	*U*^22^	*U*^33^	*U*^12^	*U*^13^	*U*^23^
Cl1	0.01319 (12)	0.03164 (15)	0.03279 (15)	0.00500 (8)	0.00046 (9)	−0.00599 (10)
O1	0.0170 (3)	0.0196 (3)	0.0230 (3)	−0.0015 (3)	−0.0012 (3)	−0.0029 (3)
C1	0.0134 (4)	0.0153 (4)	0.0143 (4)	0.0008 (3)	0.0012 (3)	0.0028 (3)
C2	0.0153 (4)	0.0186 (4)	0.0174 (4)	0.0039 (3)	0.0033 (3)	0.0016 (3)
C3	0.0202 (4)	0.0180 (4)	0.0233 (4)	0.0050 (4)	0.0013 (4)	−0.0045 (4)
C4	0.0174 (4)	0.0145 (4)	0.0138 (4)	0.0008 (3)	0.0017 (3)	0.0006 (3)
C5	0.0211 (4)	0.0158 (4)	0.0181 (4)	−0.0026 (3)	−0.0001 (3)	−0.0020 (3)
C6	0.0164 (4)	0.0208 (4)	0.0197 (4)	−0.0041 (3)	−0.0004 (3)	−0.0003 (3)
C7	0.0124 (4)	0.0203 (4)	0.0184 (4)	0.0012 (3)	0.0014 (3)	0.0000 (3)
C8	0.0138 (4)	0.0161 (4)	0.0175 (4)	0.0007 (3)	0.0015 (3)	−0.0017 (3)
C9	0.0133 (4)	0.0142 (4)	0.0134 (4)	0.0000 (3)	0.0011 (3)	0.0003 (3)

### (I) 6-Chloroindan-1-one Geometric parameters (Å, º)

**Table:**

Cl1—C7	1.7435 (11)	C4—C9	1.3947 (13)
O1—C1	1.2200 (12)	C4—C5	1.3974 (14)
C1—C9	1.4834 (13)	C5—C6	1.3913 (15)
C1—C2	1.5218 (13)	C5—H5A	0.95
C2—C3	1.5406 (15)	C6—C7	1.4054 (14)
C2—H2A	0.99	C6—H6A	0.95
C2—H2B	0.99	C7—C8	1.3880 (13)
C3—C4	1.5120 (14)	C8—C9	1.3981 (13)
C3—H3A	0.99	C8—H8A	0.95
C3—H3B	0.99		
			
O1—C1—C9	125.95 (9)	C5—C4—C3	128.81 (9)
O1—C1—C2	126.46 (9)	C6—C5—C4	119.00 (9)
C9—C1—C2	107.58 (8)	C6—C5—H5A	120.5
C1—C2—C3	106.23 (8)	C4—C5—H5A	120.5
C1—C2—H2A	110.5	C5—C6—C7	120.09 (9)
C3—C2—H2A	110.5	C5—C6—H6A	120.0
C1—C2—H2B	110.5	C7—C6—H6A	120.0
C3—C2—H2B	110.5	C8—C7—C6	122.01 (9)
H2A—C2—H2B	108.7	C8—C7—Cl1	119.08 (8)
C4—C3—C2	104.73 (8)	C6—C7—Cl1	118.90 (7)
C4—C3—H3A	110.8	C7—C8—C9	116.67 (9)
C2—C3—H3A	110.8	C7—C8—H8A	121.7
C4—C3—H3B	110.8	C9—C8—H8A	121.7
C2—C3—H3B	110.8	C4—C9—C8	122.60 (8)
H3A—C3—H3B	108.9	C4—C9—C1	109.64 (8)
C9—C4—C5	119.62 (9)	C8—C9—C1	127.76 (9)
C9—C4—C3	111.57 (8)		
			
O1—C1—C2—C3	−174.57 (9)	Cl1—C7—C8—C9	177.06 (7)
C9—C1—C2—C3	5.05 (10)	C5—C4—C9—C8	0.96 (14)
C1—C2—C3—C4	−4.48 (10)	C3—C4—C9—C8	−178.67 (9)
C2—C3—C4—C9	2.39 (11)	C5—C4—C9—C1	−179.59 (8)
C2—C3—C4—C5	−177.19 (9)	C3—C4—C9—C1	0.78 (11)
C9—C4—C5—C6	−1.02 (14)	C7—C8—C9—C4	0.33 (14)
C3—C4—C5—C6	178.54 (9)	C7—C8—C9—C1	−179.02 (9)
C4—C5—C6—C7	−0.18 (15)	O1—C1—C9—C4	175.91 (9)
C5—C6—C7—C8	1.53 (15)	C2—C1—C9—C4	−3.71 (10)
C5—C6—C7—Cl1	−177.11 (8)	O1—C1—C9—C8	−4.67 (15)
C6—C7—C8—C9	−1.57 (14)	C2—C1—C9—C8	175.71 (9)

### (I) 6-Chloroindan-1-one Hydrogen-bond geometry (Å, º)

**Table:**

*D*—H···*A*	*D*—H	H···*A*	*D*···*A*	*D*—H···*A*
C2—H2*A*···O1^i^	0.99	2.56	3.1933 (15)	121
C2—H2*B*···O1^ii^	0.99	2.59	3.5448 (14)	161

Symmetry codes: (i) *x*, −*y*+3/2, *z*+1/2; (ii) −*x*, −*y*+2, −*z*.

### (II) 6-Bromoindan-1-one Crystal data

**Table:**

C_9_H_7_BrO	*F*(000) = 416
*M**_r_* = 211.06	*D*_x_ = 1.794 Mg m^−^^3^
Monoclinic, *P*2_1_/*c*	Mo *K*α radiation, λ = 0.71073 Å
*a* = 6.489 (2) Å	Cell parameters from 9955 reflections
*b* = 17.101 (6) Å	θ = 2.4–30.6°
*c* = 7.224 (3) Å	µ = 5.19 mm^−^^1^
β = 102.964 (5)°	*T* = 125 K
*V* = 781.2 (5) Å^3^	Block, colourless
*Z* = 4	0.40 × 0.21 × 0.05 mm

### (II) 6-Bromoindan-1-one Data collection

**Table:**

Bruker APEXII CCD diffractometer	4453 independent reflections
Radiation source: fine-focus sealed tube	3600 reflections with *I* > 2σ(*I*)
Graphite monochromator	*R*_int_ = 0.046
Detector resolution: 8.3333 pixels mm^-1^	θ_max_ = 30.6°, θ_min_ = 2.4°
φ and ω scans	*h* = −9→9
Absorption correction: multi-scan (*TWINABS*; Bruker 2013)	*k* = 0→24
*T*_min_ = 0.55, *T*_max_ = 0.78	*l* = 0→10
4453 measured reflections	

### (II) 6-Bromoindan-1-one Refinement

**Table:**

Refinement on *F*^2^	0 restraints
Least-squares matrix: full	Hydrogen site location: inferred from neighbouring sites
*R*[*F*^2^ > 2σ(*F*^2^)] = 0.030	H-atom parameters constrained
*wR*(*F*^2^) = 0.152	*w* = 1/[σ^2^(*F*_o_^2^) + (0.1149*P*)^2^] where *P* = (*F*_o_^2^ + 2*F*_c_^2^)/3
*S* = 1.03	(Δ/σ)_max_ = 0.001
4453 reflections	Δρ_max_ = 1.15 e Å^−^^3^
101 parameters	Δρ_min_ = −1.15 e Å^−^^3^

### (II) 6-Bromoindan-1-one Special details

**Table:**

Experimental. BASF 0.0762 (5)
Geometry. All esds (except the esd in the dihedral angle between two l.s. planes) are estimated using the full covariance matrix. The cell esds are taken into account individually in the estimation of esds in distances, angles and torsion angles; correlations between esds in cell parameters are only used when they are defined by crystal symmetry. An approximate (isotropic) treatment of cell esds is used for estimating esds involving l.s. planes.
Refinement. Refined as a 2-component twin

### (II) 6-Bromoindan-1-one Fractional atomic coordinates and isotropic or equivalent isotropic displacement parameters (Å^2^)

**Table:**

	*x*	*y*	*z*	*U*_iso_*/*U*_eq_	
Br1	0.34409 (5)	0.45945 (2)	0.19485 (4)	0.02009 (16)	
O1	0.4929 (3)	0.12531 (11)	0.2717 (4)	0.0233 (5)	
C1	0.3083 (4)	0.14135 (17)	0.2062 (4)	0.0159 (5)	
C2	0.1260 (4)	0.08352 (17)	0.1453 (4)	0.0179 (5)	
H2A	0.1117	0.0502	0.2537	0.021*	
H2B	0.1515	0.0494	0.0419	0.021*	
C3	−0.0764 (5)	0.13304 (17)	0.0755 (4)	0.0192 (6)	
H3A	−0.1424	0.1212	−0.0589	0.023*	
H3B	−0.1806	0.1229	0.1539	0.023*	
C4	−0.0002 (4)	0.21670 (16)	0.0973 (4)	0.0154 (5)	
C5	−0.1162 (5)	0.28574 (17)	0.0538 (4)	0.0194 (5)	
H5A	−0.2646	0.2838	0.0039	0.023*	
C6	−0.0119 (4)	0.35734 (17)	0.0842 (4)	0.0178 (5)	
H6A	−0.0895	0.4045	0.0544	0.021*	
C7	0.2070 (4)	0.36019 (16)	0.1585 (4)	0.0158 (5)	
C8	0.3257 (4)	0.29262 (17)	0.2034 (4)	0.0155 (5)	
H8A	0.474	0.2947	0.2538	0.019*	
C9	0.2184 (4)	0.22114 (15)	0.1714 (4)	0.0143 (5)	

### (II) 6-Bromoindan-1-one Atomic displacement parameters (Å^2^)

**Table:**

	*U*^11^	*U*^22^	*U*^33^	*U*^12^	*U*^13^	*U*^23^
Br1	0.0266 (2)	0.0106 (2)	0.0232 (2)	−0.00221 (8)	0.00597 (15)	−0.00020 (8)
O1	0.0173 (10)	0.0158 (10)	0.0356 (13)	0.0027 (8)	0.0034 (9)	−0.0001 (9)
C1	0.0176 (11)	0.0125 (12)	0.0182 (13)	−0.0028 (9)	0.0054 (10)	−0.0013 (9)
C2	0.0192 (12)	0.0131 (12)	0.0217 (13)	−0.0026 (10)	0.0052 (10)	−0.0006 (10)
C3	0.0195 (13)	0.0168 (13)	0.0209 (13)	−0.0060 (10)	0.0035 (11)	−0.0010 (10)
C4	0.0165 (11)	0.0153 (12)	0.0144 (11)	−0.0011 (9)	0.0033 (9)	0.0006 (9)
C5	0.0157 (11)	0.0193 (13)	0.0220 (13)	0.0006 (10)	0.0015 (10)	0.0025 (10)
C6	0.0194 (12)	0.0145 (12)	0.0192 (13)	0.0043 (10)	0.0038 (10)	0.0026 (10)
C7	0.0184 (12)	0.0132 (11)	0.0161 (12)	−0.0018 (9)	0.0042 (10)	0.0002 (9)
C8	0.0155 (11)	0.0130 (13)	0.0177 (13)	−0.0011 (9)	0.0033 (10)	0.0002 (9)
C9	0.0156 (11)	0.0122 (11)	0.0151 (11)	−0.0010 (9)	0.0036 (9)	−0.0001 (9)

### (II) 6-Bromoindan-1-one Geometric parameters (Å, º)

**Table:**

Br1—C7	1.907 (3)	C4—C5	1.398 (4)
O1—C1	1.216 (3)	C4—C9	1.401 (4)
C1—C9	1.483 (4)	C5—C6	1.392 (4)
C1—C2	1.529 (4)	C5—H5A	0.95
C2—C3	1.549 (4)	C6—C7	1.402 (4)
C2—H2A	0.99	C6—H6A	0.95
C2—H2B	0.99	C7—C8	1.386 (4)
C3—C4	1.510 (4)	C8—C9	1.400 (4)
C3—H3A	0.99	C8—H8A	0.95
C3—H3B	0.99		
			
O1—C1—C9	126.1 (3)	C9—C4—C3	111.8 (2)
O1—C1—C2	126.6 (3)	C6—C5—C4	119.3 (3)
C9—C1—C2	107.3 (2)	C6—C5—H5A	120.4
C1—C2—C3	106.6 (2)	C4—C5—H5A	120.4
C1—C2—H2A	110.4	C5—C6—C7	120.4 (3)
C3—C2—H2A	110.4	C5—C6—H6A	119.8
C1—C2—H2B	110.4	C7—C6—H6A	119.8
C3—C2—H2B	110.4	C8—C7—C6	121.5 (3)
H2A—C2—H2B	108.6	C8—C7—Br1	119.5 (2)
C4—C3—C2	104.5 (2)	C6—C7—Br1	119.0 (2)
C4—C3—H3A	110.9	C7—C8—C9	117.3 (2)
C2—C3—H3A	110.9	C7—C8—H8A	121.3
C4—C3—H3B	110.9	C9—C8—H8A	121.3
C2—C3—H3B	110.9	C8—C9—C4	122.3 (2)
H3A—C3—H3B	108.9	C8—C9—C1	127.8 (2)
C5—C4—C9	119.2 (2)	C4—C9—C1	109.9 (2)
C5—C4—C3	129.0 (2)		
			
O1—C1—C2—C3	179.0 (3)	Br1—C7—C8—C9	−179.01 (19)
C9—C1—C2—C3	−0.8 (3)	C7—C8—C9—C4	−0.1 (4)
C1—C2—C3—C4	0.7 (3)	C7—C8—C9—C1	179.6 (3)
C2—C3—C4—C5	179.3 (3)	C5—C4—C9—C8	−0.1 (4)
C2—C3—C4—C9	−0.4 (3)	C3—C4—C9—C8	179.6 (2)
C9—C4—C5—C6	0.3 (4)	C5—C4—C9—C1	−179.8 (2)
C3—C4—C5—C6	−179.4 (3)	C3—C4—C9—C1	−0.1 (3)
C4—C5—C6—C7	−0.3 (4)	O1—C1—C9—C8	1.1 (5)
C5—C6—C7—C8	0.1 (4)	C2—C1—C9—C8	−179.2 (3)
C5—C6—C7—Br1	179.2 (2)	O1—C1—C9—C4	−179.2 (3)
C6—C7—C8—C9	0.0 (4)	C2—C1—C9—C4	0.6 (3)

### (II) 6-Bromoindan-1-one Hydrogen-bond geometry (Å, º)

**Table:**

*D*—H···*A*	*D*—H	H···*A*	*D*···*A*	*D*—H···*A*
C3—H3*B*···O1^i^	0.99	2.45	3.408 (4)	162
C5—H5*A*···O1^ii^	0.95	2.55	3.253 (4)	131
C2—H2*B*···Br1^iii^	0.99	3.05	3.898 (3)	145

Symmetry codes: (i) *x*−1, *y*, *z*; (ii) *x*−1, −*y*+1/2, *z*−1/2; (iii) *x*, −*y*+1/2, *z*−1/2.
